# Incorporating Technology in the Assessment of Financial Decision Making – Preliminary Findings from a Simulated Online Credit Card Task from Older Adults in the United States

**DOI:** 10.1002/alz70857_105755

**Published:** 2025-12-25

**Authors:** Preeti Sunderaraman, Madison Bouchard‐Liporto, Silvia Chapman, Sandra Rizer, Masanao Yajima, Siqi Zhao, Stephanie Cosentino

**Affiliations:** ^1^ Framingham Heart Study, Boston University Chobanian & Avedisian School of Medicine, Boston, MA, USA; ^2^ Boston University Chobanian & Avedisian School of Medicine, Boston, MA, USA; ^3^ Boston University Chobanian& Avedisian School of Medicine, Boston, MA, USA; ^4^ Columbia University Irving Medical Center, New York, NY, USA; ^5^ Department of Mathematics and Statistics, Boston University, Boston, MA, USA; ^6^ The Taub Institute for Research on Alzheimer's Disease and the Aging Brain, Columbia University, New York, NY, USA

## Abstract

**Background:**

Credit card use is ubiquitous globally and across all age groups. In 2023, around $10 billion was lost in credit card related scams and fraud in the United States. Older adults are at increased risk for such financial loss due to cognitive decline and increased dementia risk. Still, tools to assess such online money management (OMM) skills are limited. This study sought to validate the factor structure of a novel, simulated OMM credit card task designed to replicate real‐world scenarios.

**Method:**

The OMM task was conceptualized through collaboration among neuropsychologists, economists, computer scientists, and gerontologists. Data were collected from 169 participants (M age (yrs) = 76.78; *SD* = 4.26, *M* education (yrs) = 21.86, *SD* = 1.88, 69.6% female, 90% White 7% Black). The OMM task comprises of various components, including online navigation (ON) which involves logging into a simulated credit card account to download a statement, and content‐focused tasks such as simple literacy (SL) which requires clicking on answers in response to questions about the statement, and statement monitoring (SM) which requires identifying erroneous transactions. Confirmatory factor analyses (CFA) were performed to test the hypothesized three factors: ON, SL, and SM. Metrics for each factor included number of clicks and time taken to complete each task.

**Result:**

The CFA results supported the hypothesized three‐factor model, showing good model fit indices (chi‐square = 5.29, *p* =  0.51; RMSEA = 0; CFI = 1; SRMR = 0.03). Standardized factor loadings were acceptable for all 3 factors and varied from 0.41 to 1.60. Number of clicks exhibited relatively weak loadings, while completion time displayed stronger loadings on all the three factors, especially for SM.

**Conclusion:**

Findings suggest that the time required to complete tasks is a relatively more reliable metric for assessing OMM skills than number of clicks. The validated factors align with the conceptualized components of the OMM task, which shows promise for use in clinical assessments. To examine the generalizability of these findings, future studies should examine the task in racially and clinically diverse populations.

